# Growth kinetics of multiple *Acinetobacter baumannii* resistotype after meropenem-based antibiotic combination exposure

**DOI:** 10.12688/f1000research.122221.2

**Published:** 2022-11-28

**Authors:** Erizka Rivani, Pepy Dwi Endraswari, Agung Dwi Wahyu Widodo

**Affiliations:** 1 Department of Microbiology, Faculty of Medicine, Sriwijaya University, Palembang, South Sumatera, 30114, Indonesia; 2 Department of Microbiology, Faculty of Medicine, Airlangga University, Surabaya, East Java, 60115, Indonesia; 3 Clinical Microbiology Department, Dr. Soetomo General Academic Hospital, Surabaya, East Java, 60286, Indonesia

**Keywords:** Acinetobacter baumannii, antibiotic combinations, time-kill, meropenem, ampicillin-sulbactam, amikacin

## Abstract

**Background:** Carbapenems are the treatment of choice for multidrug-resistant (MDR) and extensively drug-resistant (XDR) *Acinetobacter baumannii* infections, but the emergence of carbapenem-resistant *A. baumannii* (CRAB) has rendered it ineffective in the vast majority of cases. Combination therapy has grown in popularity over the last decade; this study aims to analyze *A.baumannii* growth kinetics after exposure to meropenem and ampicillin-sulbactam compared with meropenem and amikacin antibiotic combinations in clinically relevant concentrations.

**Methods:** This experimental laboratory study was conducted on the *A. baumannii* ATCC 19606 isolate and three clinical isolates that were intermediate or resistant to tested antibiotics. Meropenem and ampicillin-sulbactam, as well as meropenem and amikacin, were tested at four different concentrations against isolates. Turbidity measurements were taken at predetermined time points of 0, 1, 2, 4, 6, 8, and 24 hours following exposure; bacterial concentration was enumerated using the agar plate method, with the results plotted in a time-kill curve.

**Results:** A bactericidal effect was achieved in isolates that were intermediate to ampicillin-sulbactam and resistant to meropenem after the administration of meropenem and ampicillin-sulbactam combination with a concentration of 4 µg/ml and 16/8 µg/ml, respectively. The combination of meropenem and ampicillin-sulbactam demonstrated bacteriostatic activity against isolates that were resistant to both antibiotics. Isolates treated with resistant antibiotics showed an increased growth rate compared to the growth control.

**Conclusion:** The combination of meropenem and ampicillin-sulbactam could be a promising combination therapy in treating CRAB infections. The mechanism and degree of antibiotic resistance in the isolates affect the efficacy of antibiotic combinations; further research is needed to corroborate the findings of this study.

## Introduction

*Acinetobacter baumannii* is a Gram-negative rod that garners attention due to its role as a primary pathogen in healthcare-associated infections with a broad spectrum of antibiotic resistance ^1, 2^. Carbapenems are the preferred treatment for multidrug-resistant (MDR) *A. baumannii* infections. However, treatment options have dwindled due to high isolation rates of extensively drug-resistant (XDR) *A. baumannii* with concurrent carbapenem resistance ^3, 4^.

The discovery of new antibiotics is critical for treating MDR and XDR *A. baumannii* infections. Nevertheless, antibiotic studies take a long time to complete and are difficult to implement in developing countries with limited access to the latest antibiotics. The alternative strategy that has gathered the most interest is antibiotic combination therapy, which is theoretically supposed to boost antibiotic effectiveness compared to single antibiotics ^5– 7^.

In studies evaluating antibiotic combinations, isolates that are susceptible to at least one of the regimens are frequently used, whereas many *A. baumannii* clinical isolates frequently lack susceptibility to any antibiotic ^5, 8^. Additionally, because the antibiotic concentrations used in studies are typically multiple times of minimum inhibitory concentration (MIC) and are difficult to achieve during the administration of therapeutic antibiotic doses, the clinical application of study results is complicated ^5, 9– 11^.

Meropenem is one of the few remaining low-toxicity treatment options for MDR and XDR *A. baumannii* infections ^12, 13^. Sulbactam is a beta-lactamase inhibitor with intrinsic activity against *A. baumannii*, whilst amikacin is an aminoglycoside with relatively maintained efficacy against multidrug-resistant Gram-negative bacteria, including *A. baumannii* ^14– 17^. Ampicillin-sulbactam and amikacin are two antibiotics that are available and easy to obtain in Indonesia. A sole sulbactam regimen is not available; it is marketed in conjunction with ampicillin or cefoperazone. Ampicillin-sulbactam formulations were chosen because of the availability of breakpoints in CLSI M100 2022 and technical considerations such as affordability and convenience of access to the antibiotics.

 Numerous *in vitro* studies have demonstrated synergy between meropenem and ampicillin-sulbactam as well as meropenem and amikacin; thus, this study aimed to compare the growth kinetics of various *A. baumannii* strains exposed to these two antibiotic combinations at clinically relevant concentrations ^18– 23^.

## Methods

### Study design

Experiments were conducted on two MDR, one XDR clinical isolates from Clinical Microbiology Laboratorium Dr. Soetomo General Academic Hospital, and one standard reference isolate (ATCC *A. baumannii* 19606 KWIK-STIK ^TM^ Microbiologics). All clinical isolates are meropenem resistant, conforming to the Clinical and Laboratory Standard Institute (CLSI) 2022 breakpoint for *A. baumannii* (MIC >8 μg/ml as determined by an automatic susceptibility test using BD Phoenix® ID/AST instrument). MDR-1 is resistant to meropenem and amikacin (MIC >32 μg/ml) but is intermediate to ampicillin-sulbactam (MIC 16/8 μg/ml); MDR-2 is resistant to meropenem and ampicillin-sulbactam (MIC >16/8 μg/ml) but is intermediate to amikacin (MIC 32 μg/ml). XDR exhibited resistance to all antibiotics tested.

### Ethical considerations

This study was reviewed by the Ethics Committee of the Faculty of Medicine, Airlangga University (0758/LOE/301.4.2/I/2022).

### Procedure

Drug concentrations were selected based on the CLSI breakpoint value for the susceptible category of tested antibiotics as it represents clinically achievable concentrations of drugs in human plasma following standard dosing. Fresh stocks of each antibacterial were prepared on the day of the experiment to achieve 0.5 MIC + 0.5 MIC, 1 MIC + 1 MIC, 2 MIC + 2 MIC, and 2 MIC + 0.5 MIC of meropenem + ampicillin-sulbactam and meropenem + amikacin (Sigma). Prior to the time-kill assay experiment, strains were subcultured onto blood agar (Oxoid CM0055 Blood Agar Base supplemented with 5% sheep blood) and incubated for 24 hours at 35°C. Mid-log phase growth suspension was obtained by inoculating isolated colony into cation-adjusted Mueller-Hinton broth (Oxoid CM0405 Mueller-Hinton Broth base) followed by 4 hours of incubation at 35°C. Static time-kill experiments were performed in sextuplicates on separate days at an initial inoculum of 6×10 ^5^ CFU/ml with the combined antibiotic concentrations in the glass tube, incubated at 35°C. Samples were collected at 0, 1, 2, 4, 6, 8, and 24 h, measured for turbidity by nephelometer (BD PhoenixSpec ^TM^ Nephelometer), serially diluted in saline, plated on Mueller-Hinton agar (Oxoid CM 0337 Muelle-Hinton Agar base), and counted after 24 h of incubation for viable-cell counting. Enumeration was performed manually after 24 hours of incubation at 35°C. The limit of detection (LOD) was 10 ^2^ CFU/ml. In the meantime, a control experiment was carried out simultaneously with the same procedure without antibiotic addition. Bactericidal activity was assessed as a ≥ 3 log _10_ reduction in a colony-forming unit (CFU)/mL over the period measured. Regrowth was defined as an initial decrease of turbidity or colony count followed by an escalation in the subsequent measurement hour.

## Results

The turbidity and colony count data did not follow a normal distribution (Shapiro-Wilk value 0.000). There were significant differences in mean turbidity between isolates of ATCC 19606, MDR-1, MDR-2, and XDR at 2, 4, 6, 8, and 24 hours following antibiotic exposure (p<0.05; Wilcoxon; CI 95%). There were significant differences in the mean colony count between isolates of ATCC 19606, MDR-1, MDR-2, and XDR at 6, 8, and 24 hours following exposure, (p = 0.001, p = 0.01, and p = 0.000; Wilcoxon; CI 95%). The full turbidity and colony count data can be found under *Underlying Data* ^24^.

**Figure 1.**
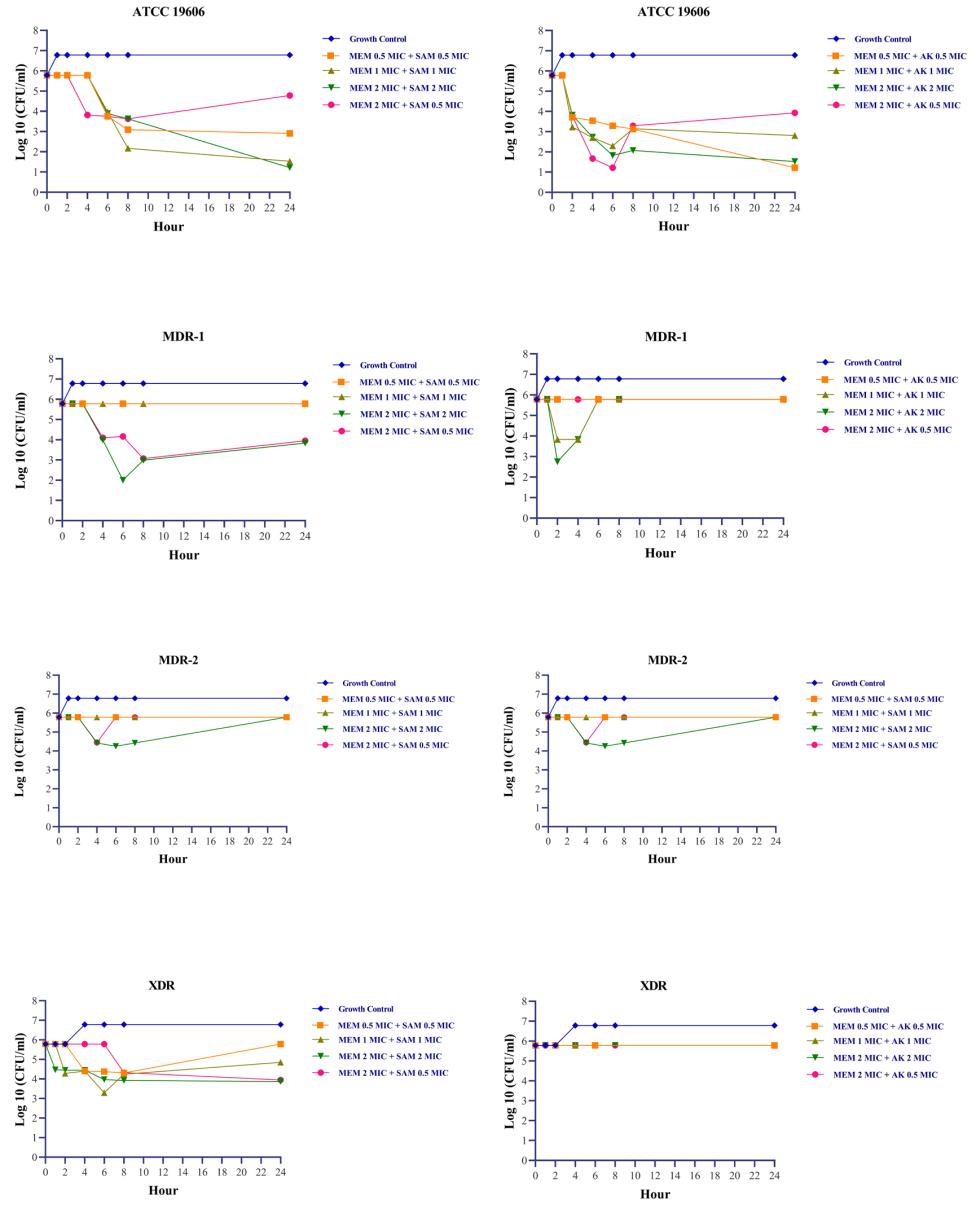
Colony count fluctuations of *Acinetobacter baumannii* following exposure with meropenem + ampicillin sulbactam and meropenem + amikacin combination ^24^. MEM: *meropenem,* SAM: *ampicillin-sulbactam,* AK: *amikacin,* MIC: *minimum inhibitory concentration. Acinetobacter baumannii’*s MIC based on CLSI 2022 susceptible breakpoint: Meropenem 2 µg/ml, Ampicillin-Sulbactam 8/4 µg/ml, Amikacin 16 µg/ml.

**Figure 2.**
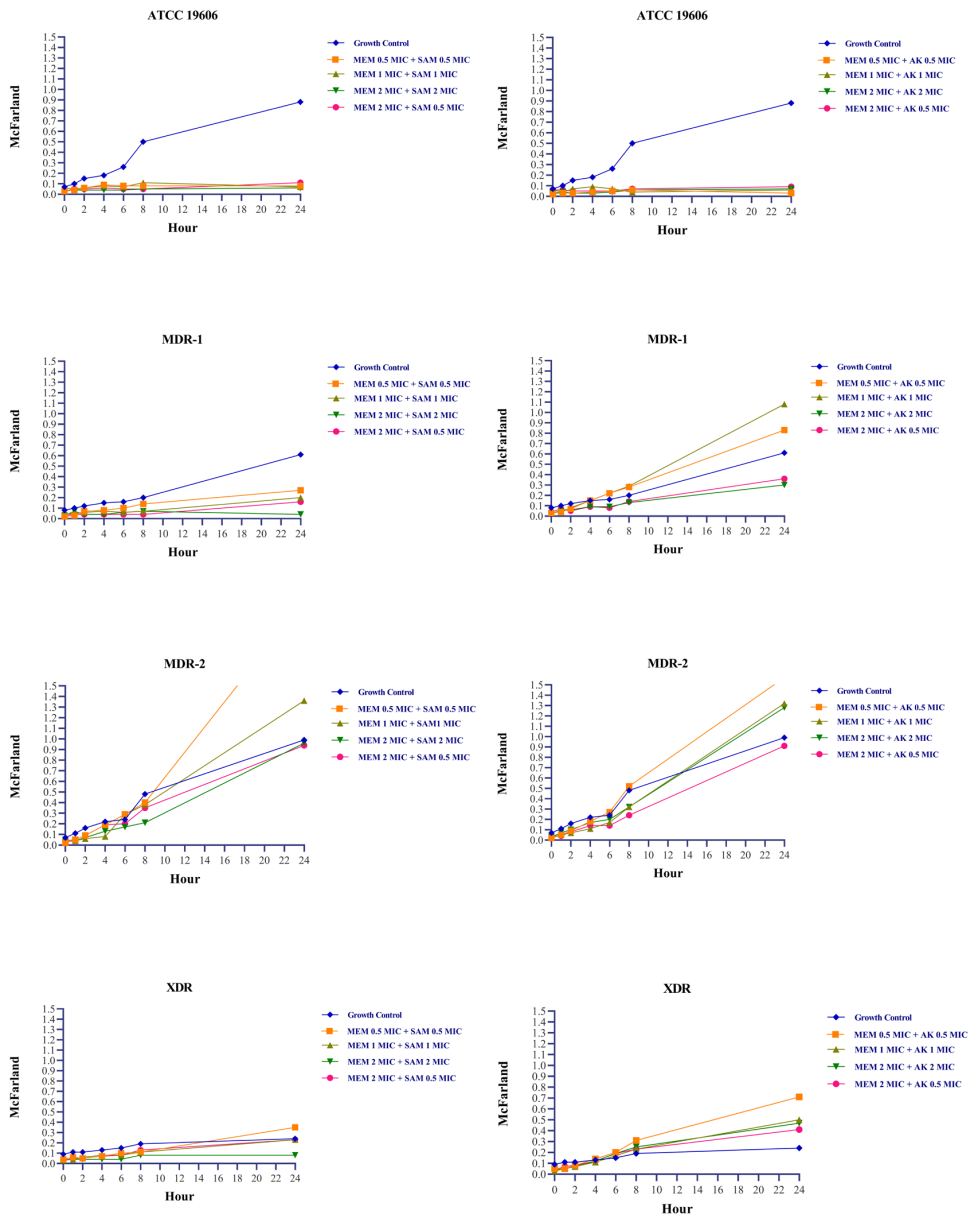
Turbidity fluctuations in *Acinetobacter baumannii* suspension following exposure with meropenem + ampicillin-sulbactam and meropenem + amikacin combination ^24^. MEM: *meropenem,* SAM: *ampicillin-sulbactam,* AK: *amikacin,* MIC: *minimum inhibitory concentration. Acinetobacter baumannii’*s MIC based on CLSI 2022 susceptible breakpoint: Meropenem 2 µg/ml, Ampicillin-Sulbactam 8/4 µg/ml, Amikacin 16 µg/ml.

Exposures to meropenem and ampicillin-sulbactam yield encouraging results. In the MDR-1 isolate, which was resistant to carbapenem and intermediate to ampicillin-sulbactam, the bactericidal effect of meropenem and ampicillin-sulbactam was achieved at a 2 MIC + 2 MIC concentration, respectively ( Figure 1). During 0–24 hours, concentrations of 0.5 MIC + 0.5 MIC, 1 MIC + 1 MIC, and 2 MIC + 0.5 MIC were able to sustain growth under the rate of growth control, as demonstrated by turbidity measurements. However, the turbidity was approximately indistinguishable at 48 hours ( Figure 2). Changes in the number of colonies could not be observed at 0.5 MIC + 0.5 MIC and 1 MIC + 1 MIC concentration due to high colony count results. Exposure to a 2 MIC + 0.5 MIC concentration caused a transient inhibitory effect for up to 4 hours, but regrowth occurred at the hour of measurement thenceforth.

MDR-2 isolate (isolate resistant to meropenem and ampicillin-sulbactam) treated with meropenem and ampicillin-sulbactam combination at concentration equal to or less than the MIC demonstrated higher turbidity compared to positive growth control after 24 and 48 hours. At a concentration twice the MIC, there is a reduction in colony count after four hours, followed by regrowth. During post-exposure monitoring, XDR isolate exposed to meropenem and ampicillin-sulbactam did not show any signs of regrowth, except at a concentration of 1 MIC + 1 MIC, where regrowth occurred at 8 and 24 hours ( Table 1).

**Table 1.** Antibiotic combination activity against *Acinetobacter baumannii* isolates.

Isolate	Antibiotic	Concentration ^a^	Activity ^b^	Δ Log 10 ^c^	*Regrowth ^d^*	Turbidity measurement higher than growth control ^e^
ATCC 19606	MEM + SAM	½ MIC + ½ MIC	Bacteriostatic	2.87	No	No
		1 MIC + 1 MIC	Bactericidal	4.25	No	No
		2 MIC + 2 MIC	Bactericidal	4.56	No	No
		2 MIC + ½ MIC	Bacteriostatic	2.15	Yes	No
	MEM + AK	½ MIC + ½ MIC	Bactericidal	4.56	Yes	No
		1 MIC + 1 MIC	Bactericidal	3.48	Yes	No
		2 MIC + 2 MIC	Bactericidal	4.26	Yes	No
		2 MIC + ½ MIC	Bactericidal	4.56	Yes	No
MDR 1	MEM + SAM	½ MIC + ½ MIC	Bacteriostatic	0	Yes	No
		1 MIC + 1 MIC	Bacteriostatic	0	Yes	No
		2 MIC + 2 MIC	Bactericidal	3.78	Yes	No
		2 MIC + ½ MIC	Bacteriostatic	2.71	Yes	No
	MEM + AK	½ MIC + ½ MIC	Bacteriostatic	0	Yes	Yes, since hour 6 after exposure
		1 MIC + 1 MIC	Bacteriostatic	1.95	Yes	Yes, since hour 6 after exposure
		2 MIC + 2 MIC	Bacteriostatic	2.83	Yes	No
		2 MIC + ½ MIC	Bacteriostatic	0	Yes	No
MDR 2	MEM + SAM	½ MIC + ½ MIC	Bacteriostatic	0	Yes	Yes, at hour 24 after exposure
		1 MIC + 1 MIC	Bacteriostatic	0	Yes	Yes, at hour 24 after exposure
		2 MIC + 2 MIC	Bacteriostatic	1.53	Yes	No
		2 MIC + ½ MIC	Bacteriostatic	1.33	Yes	No
	MEM + AK	½ MIC + ½ MIC	Bacteriostatic	0	Yes	Yes, since hour 8 after exposure
		1 MIC + 1 MIC	Bacteriostatic	0	Yes	Yes, at hour 24 after exposure
		2 MIC + 2 MIC	Bacteriostatic	1.79	Yes	Yes, at hour 24 after exposure
		2 MIC + ½ MIC	Bacteriostatic	0	Yes	No
XDR	MEM + SAM	½ MIC + ½ MIC	Bacteriostatic	1.47	No	Yes, at hour 24 after exposure
		1 MIC + 1 MIC	Bacteriostatic	2.48	Yes	No
		2 MIC + 2 MIC	Bacteriostatic	1.91	No	No
		2 MIC + ½ MIC	Bacteriostatic	1.83	No	No
	MEM + AK	½ MIC + ½ MIC	Bacteriostatic	0	Yes	Yes, since hour 6 after exposure
		1 MIC + 1 MIC	Bacteriostatic	0	Yes	Yes, since hour 6 after exposure
		2 MIC + 2 MIC	Bacteriostatic	0	Yes	Yes, since hour 6 after exposure
		2 MIC + ½ MIC	Bacteriostatic	0	Yes	Yes, since hour 6 after exposure

ATCC: *American Type Culture Collection,* MDR: *multidrug-resistant,* XDR: *extensively drug-resistant*, MEM: *meropenem*, SAM: *ampicillin-sulbactam,* AK: *amikacin,* MIC: *minimum inhibitory concentration* ^a^: Meropenem MIC = 2 μg/ml; Ampicillin-Sulbactam MIC: 8/4 μg/ml; Amikacin MIC: 16 μg/ml ^b^: Bactericidal: ≥ 3 log _10_ reduction in a colony-forming unit (CFU)/ml over the period measured. Bacteriostatic: < 3 log _10_ reduction in a colony-forming unit (CFU)/mL over the period measured (compared to initial measurement of tested isolate) ^c^: **Δ Log 10: Log 10 of the total colony-forming unit (CFU/ml) reduction over the measurement time** (compared to initial measurement of tested isolate) ^d^: Regrowth: initial decrease of turbidity or colony count followed by an escalation in the subsequent measurement hour ^e^: Comparison of the colony count between the treatment group and growth control group of isolate. Growth control: isolate without antibiotic combination exposure

Meropenem and amikacin had no bactericidal impact on intermediate and drug-resistant isolates; hence on all clinical isolates of *A. baumannii* in this study. The most significant reduction in the number of bacteria was observed following exposure to 2 MIC and 2 MIC; however, these concentrations had no effect on the number of colonies in XDR isolates when compared to the number of colonies at 0 hours measurement.

## Discussion

This investigation discovered regrowth in clinical isolates from nearly all exposure groups. Regrowth is influenced by various factors related to the concentration of antibiotics and bacterial inoculum, as well as the susceptibility of bacteria ^25^. Regrowth may occur when bacterial growth is not fully inhibited by exposure to antibiotics (due to insufficient antibiotic concentration or a resistant bacterial strain) ^26^. Persistent/resistant bacterial subpopulations can also be inferred from time-kill curve regrowth ^27– 29^. Antibiotic degradation in the test suspension also plays a role; decreased active antibiotic amount during the final hours of testing may render inhibition ineffective, allowing regrowth to occur ^30^.

Meropenem and ampicillin-sulbactam are time-dependent beta-lactam antibiotics ^31^. The synergism may be due to the distinct penicillin-binding proteins (PBP) binding mechanisms, hence enhancing the activity of beta-lactams in bacteria ^32^. Meropenem has a high affinity for PBP 2, PBP 3, PBP 1a, and PBP 1b, ampicillin has a high affinity for PBP 4, and sulbactam has a high affinity for PBP 1 and PBP 3 ^33, 34^. The downregulation of native and subsequent synthesis of altered PBPs is one of the mechanism behind *A. baumannii's* resistance to beta-lactam antibiotics ^35– 37^. In addition to its simultaneous action on PBP, sulbactam's beta-lactam inhibitory activity can boost meropenem's affinity and, consequently, activity ^38, 39^. Numerous investigations have demonstrated that subinhibitory concentrations of beta-lactam antibiotics can alter the shape of bacteria's cell walls ^40^. In theory, it has the potential to augment the intake of other antibiotics ^41^.

Meropenem in combination with ampicillin-sulbactam at a concentration twice the MIC was bactericidal against isolates intermediate to ampicillin-sulbactam. Moreover, it had a lower rate of regrowth than the meropenem and amikacin exposure groups. Differences in resistance levels are believed to have an effect on the efficiency of antibiotic combinations ^42– 44^. It should be anticipated that the distinct resistance mechanisms held by various strains resulted in different responses to combination antibiotic exposure ^20, 45, 46^.

Additionally, this study found that isolates treated at sub-MIC concentrations of antibiotics had a higher colony count than the growth control group. This finding merits additional investigation to ascertain the underlying mechanism. Antibiotics have a selection and inducer effect on antibiotic resistance, which demonstrates the importance of using them prudently.

## Conclusions

Meropenem in combination with ampicillin-sulbactam at a concentration twice the MIC was bactericidal against isolates resistant to meropenem and intermediate to ampicillin-sulbactam. Meropenem and ampicillin-sulbactam in combination demonstrated bacteriostatic activity against isolates resistant to both antibiotics. Meropenem and amikacin in combination had no bactericidal effect on isolates that were either intermediate or resistant to meropenem and amikacin. Combined administration of meropenem and ampicillin-sulbactam can be considered in cases of *A. baumannii* infection that is not susceptible to any antibiotics. Higher doses show better results and should be attempted when clinical circumstances allow.

## Data Availability

Figshare: Colony Count and Turbidity Data from Time-Kill Assay of Acinetobacter baumannii exposed to Meropenem-based Antibiotic Combinations. https://doi.org/10.6084/m9.figshare.20024270.v3 ^24^. This project contains the following underlying data: Colony Count Data.csv Turbidity Data.csv Data are available under the terms of the Creative Commons Zero "No rights reserved" data waiver (CC0 1.0 Public domain dedication).
