# Supplementary material for: Growth kinetics of multiple Acinetobacter baumannii resistotype after meropenem-based antibiotic combination exposure
Source: F1000Res. 2022 Nov 28;11:762. Originally published 2022 Jul 8. [Version 2] doi: 10.12688/f1000research.122221.2 (PMC9723411; doi:10.12688/f1000research.122221.2)
Supplement: Peer review history

# Peer review: Reviewer response for version 2

**DOI:** 10.5256/f1000research.140132.r156794

**Published:** 2022-12-05

**Authors:** Sarunyou Chusri, Arnon Chukamnerd

**Affiliations:** 1 Division of Infectious Diseases, Prince of Songkla University, Songkhla, Thailand; 2 Faculty of Medicine, Prince of Songkla Univiversity, Hat Yai, Thailand

The author responded to all comments. 

 No further requirement is needed.

Is the work clearly and accurately presented and does it cite the current literature?

Yes

If applicable, is the statistical analysis and its interpretation appropriate?

Yes

Are all the source data underlying the results available to ensure full reproducibility?

Partly

Is the study design appropriate and is the work technically sound?

Yes

Are the conclusions drawn adequately supported by the results?

Partly

Are sufficient details of methods and analysis provided to allow replication by others?

Partly

Reviewer Expertise:

Microbiology and epidemiology

We confirm that we have read this submission and believe that we have an appropriate level of expertise to confirm that it is of an acceptable scientific standard.

# Peer review: Reviewer response for version 1

**DOI:** 10.5256/f1000research.134184.r149383

**Published:** 2022-10-07

**Authors:** Ardiana Kusumaningrum

**Affiliations:** 1 Department of Microbiology, Faculty of Medicine, Clinical Microbiology Medicine Staff Group, Universitas Indonesia Hospital, Universitas Indonesia, Depok, Indonesia

In general, this article provides important in vitro data regarding the potential use of antibiotics combination in *Acinetobacter baumannii* infection management. The research method used is appropriate.

 However, there are several issues that need to be considered:

 1. Is there any preliminary examination to ensure that the antibiotic concentration used is as expected, at the beginning and also the end of observation time?

 2. How did the author confirm that the bacterial isolates being tested were in exponential growth period/log phase?

 3. Is it possible to conduct duplo testing to increase the strength of study method?

 4. Figure 1 --> is there any missing line on the picture? For example, at XDR colony count fluctuations, there are only 2 lines observed (growth control and MEM 0,5MIC + AK 0,5MIC)

Is the work clearly and accurately presented and does it cite the current literature?

Yes

If applicable, is the statistical analysis and its interpretation appropriate?

I cannot comment. A qualified statistician is required.

Are all the source data underlying the results available to ensure full reproducibility?

Yes

Is the study design appropriate and is the work technically sound?

Partly

Are the conclusions drawn adequately supported by the results?

Partly

Are sufficient details of methods and analysis provided to allow replication by others?

Partly

Reviewer Expertise:

antimicrobial resistance, MDRO

I confirm that I have read this submission and believe that I have an appropriate level of expertise to confirm that it is of an acceptable scientific standard.

# Response

**Published:** 2022-10-21

**Authors:** Erizka Rivani

**Affiliations:** Airlangga University, Indonesia

Dear Dr. Ardiana,

 My co-authors and I were pleased to receive your response and the opportunity to resubmit a revised version of this manuscript. We attempt to respond to reviewer questions with relevant information obtained from our research.

 1. Is there any preliminary examination to ensure that the antibiotic concentration used is as expected at the beginning and end of observation time?

- Thank you for the question.
- There were two preliminary trials conducted before this study. The first preliminary trial was carried out to determine the appropriate colony measurement method for the test isolates exposed to the selected antibiotics for this study. The second preliminary study was carried out to determine the time required by the test isolates to reach the log phase of growth. 
- We did not conduct a preliminary trial to ascertain the concentration of the test antibiotic because antibiotic exposure was performed for 24 hours (additional measurements were taken at 48 hours to collect post-antibiotic exposure data), which is comparable to the duration of antibiotic susceptibility tests conducted in clinical microbiology laboratory with antibiotic powders that were subjected to routine quality control.

2. How did the author confirm that the bacterial isolates tested were in the exponential growth period/log phase?

- Thank you for drawing attention to this.
- In the preliminary test, isolates were grown without antibiotic treatment in liquid media. This test is designed to determine the time required for the test isolate to reach the log phase under identical conditions to the actual test. After transferring isolated colonies from solid to liquid media, turbidity measurements and colony growth calculations were undertaken every 30 minutes. The data obtained was therefore plotted on a growth curve. According to the preliminary test results, all isolates entered the log phase after two hours of incubation, and six hours later, they began to reach the stationary phase. Therefore, in the actual experiment, isolated colonies were cultured in liquid media for four hours prior to the time-kill test (at the mid-log phase).

3. Is it possible to conduct duplo testing to increase the strength of study method?

- Thank you for the comment.
- The tests were carried out six times over the course of two days. On the first day of the trial, three replications were performed. On the second day, three additional replications were conducted, bringing the total number of replications to six. This experiment was repeated six times with four test isolates treated with two types of combination antibiotics at four different concentrations on each combination.

4. Figure 1 --> is there any missing line on the picture? For example, at XDR colony count fluctuations, there are only 2 lines observed (growth control and MEM 0,5MIC + AK 0,5MIC)

- We thank you for bringing this to our attention.
- Several lines in the figure are joined because they have the same value, so the end result gives the impression that some figures are missing lines (there are just two lines instead of five). We shall attempt to revise the graphic to ensure its meaning is more evident.

# Peer review: Reviewer response for version 1

**DOI:** 10.5256/f1000research.134184.r147379

**Published:** 2022-08-19

**Authors:** Sarunyou Chusri

**Affiliations:** 1 Division of Infectious Diseases, Prince of Songkla University, Songkhla, Thailand

The brief report by Erizka et al. demonstrated the bacteriostatic and bactericidal activities of meropenem in combination with ampicillin-sulbactam and amikacin. The finding showed that the meropenem + ampicillin-sulbactam provided the good activity against these isolates, which might be considered as an alternative treatment for *A* *.* *baumannii* infection, especially MDR and XDR strains. However, I have some comments to be addressed. 

**Major comments **

1. Page 6, in the results section: “XDR isolates which were also resistant to meropenem and ampicillin-sulbactam, did not show any regrowth phenomena during post-exposure monitoring except at a concentration of 1 MIC + 1 MIC, where regrowth occurred at 8 and 24 hours” – please verify these results again. I found that regrowth occurred not only at a concentration of 1 MIC + 1 MIC but also at a concentration of 0.5 MIC + 0.5 MIC and other concentrations. In addition, only 1 isolate of XDR was included in the study. Why did the authors write “XDR isolates” in this sentence? 
2. Page 6, in the results section: “Meropenem and amikacin had no bactericidal impact on intermediate and drug-resistant isolates; hence on all clinical isolates of *A* *.* *baumannii* in this study.” – according to Figure 1 and Table 1, not all concentrations of MEM + AK have no bactericidal effect on the clinical isolates. At twice MIC of this combination against MDR-1, it seems to provide ≥ 3 log _10_ reductions, which was considered to have bactericidal activity. Please rephrase this sentence. 
3. Please carefully verify the combination activity (bactericidal and bacteriostatic) in Table 1, because there are some incorrect results. 
  - The authors reported the bacteriostatic activity in the MEM 0.5 MIC + SAM 0.5 MIC against ATCC 19606 isolate, the MEM 2 MIC + SAM 0.5 MIC against MDR-1 isolate, the MEM 2 MIC + AK 2 MIC against MDR-1, and the MEM 1 MIC + SAM 1 MIC against XDR, whereas it seems to provide ≥ 3 log _10_ reductions (bactericidal), compared to the growth control (Figure 1). 
  - “Colony count higher than growth control ^d^” – did the authors mean “Turbidity higher than growth control ^d^”? Because the results seem to be received from Figure 2. If yes, please change the title and the description of this column. 
4. The authors should recreate the supplementary tables (Colony Count Data and Turbidity Data) in data availability (underlying data). The information should be clearly and easily understood. 

**Minor comments **

1. Page 1, in the abstract section, in a part of the results: “… that were intermediate to ampicillin sulbactam and …” – a hyphen between ampicillin sulbactam was missed. 
2. Page 2, in the keywords section: “Acinetobacter baumannii” should be italic. 
3. Page 3, in the methods section, in a part of the study design: “… ATCC A.baumannii 19606 …” – a space between genus and species was missed. 
4. Page 3, in the methods section, in a part of the study design: “… (CLSI) 2022 breakpoint for A.baumannii …” – again, a space between genus and species was missed, and please check this point throughout the manuscript. 
5. Page 3, in the methods section, in a part of the procedure: “… in a colony-forming unit (CFU)/mL over …” – “mL” should be replaced by “ml” as same as the other part of the manuscript. 
6. Page 4, in the results section: “In MDR 1 isolates, sc. MDR isolates that …” – what does the “sc.” mean? 
7. Page 4, in the results section: “In MDR 1 isolates, sc. MDR isolates that were both carbapenem-resistant and intermediate to ampicillin-sulbactam” – please rephrase this sentence. 
8. Page 5, in the results section: “At 24 and 48 hours, MDR 2 isolates (isolates resistant to meropenem and ampicillin-sulbactam) …” – did the authors mean the isolate that was coded as “MDR 2”? If yes, it might be better to change the code of “MDR 1” and “MDR 2” isolates. Because these codes make confusion between the code and the number of the isolates. As shown in this case, it also means 2 isolates of MDR bacteria. The authors could use other codes such as “MDR-1” and “MDR-2”. 
9. Pages 6 - 7, in the results section: “… these concentrations had no effect on the number of colonies in XDR isolates when compared to the number of colonies at 0 hours measurement.” – according to Figure 1, the authors should also specify the type of antibiotic combination, because this phenomenon was only found in XDR against MEM + AK combination, but not XDR against MEM + SAM. 
10. Page 7, in the discussion section: “… sulbactam has a high affinity for PBP 1 and 3” could be replaced by “… sulbactam has a high affinity for PBP 1 and PBP 3”. 
11. Page 7, in the discussion section: “Numerous investigations have demonstrated that subinhibitory concentrations of beta-lactam antibiotics can alter the shape of bacteria’s cell walls” – please provide some references for this sentence. 
12. Figures 1 and 2: the color and the shape represented the results of “Growth Control” were similar to the results of “2 MIC + 0.5 MIC”. In the case of turbidity fluctuations in the MEM + AK combination against XDR isolate (Figure 2), the color and the shape represented the results of “Growth Control” were exactly the same as the results of “2 MIC + 0.5 MIC”. The authors probably use another color and shape (such as the star shape) to represent the results of “Growth Control”. 
13. How many replications did the authors performed for time-kill assay? 
14. According to the description in Figure 1, Figure 2, and Table 1, why did the MICs not represent the exact MICs, but it represents the MIC at the susceptible breakpoint from CLSI guideline? 

Is the work clearly and accurately presented and does it cite the current literature?

Yes

If applicable, is the statistical analysis and its interpretation appropriate?

Yes

Are all the source data underlying the results available to ensure full reproducibility?

Partly

Is the study design appropriate and is the work technically sound?

Yes

Are the conclusions drawn adequately supported by the results?

Partly

Are sufficient details of methods and analysis provided to allow replication by others?

Partly

Reviewer Expertise:

Microbiology and epidemiology

I confirm that I have read this submission and believe that I have an appropriate level of expertise to confirm that it is of an acceptable scientific standard, however I have significant reservations, as outlined above.

# Response

**Published:** 2022-10-21

**Authors:** Erizka Rivani

**Affiliations:** Airlangga University, Indonesia

Dear dr. Chusri,

 My co-authors and I were pleased to receive your response and the opportunity to resubmit a revised version of this manuscript. We would like to thank you for providing your constructive and detailed review comments on our manuscript. 

 We have attempted to fully address comments in the revised manuscript; the reviewer's original comments are listed below, followed by our response to each comment. Edited text in the attached revised manuscript is visible as tracked changes under the markup mode of Microsoft Word that we've sent to Editor.

 All authors have read and approved the revised manuscript. We hope our resubmission is now suitable for acceptance, and we look forward to hearing from you.

**Major Revision**

 1. Page 6, Result

*“XDR isolates which were also resistant to meropenem and ampicillin-sulbactam, did not show any regrowth phenomena during post-exposure monitoring except at a concentration of 1 MIC + 1 MIC, where regrowth occurred at 8 and 24 hours”*

 Please verify these results again. I found that regrowth occurred not only at a concentration of 1 MIC + 1 MIC but also at a concentration of 0.5 MIC + 0.5 MIC and other concentrations.

- Thank you for this observation. The second and third paragraphs of the Results section describe the measurement of the first antibiotic combination exposures, meropenem and ampicillin-sulbactam. The fourth paragraph discussed the results of meropenem and amikacin exposure. In XDR isolates exposed to meropenem and ampicillin-sulbactam, regrowth only occurred at a concentration of 1 MIC + 1 MIC. In XDR isolates exposed to meropenem and amikacin, regrowth did occur in all concentration groups.To clarify this conclusion, we attempt to rearrange the sentences.

Only 1 isolate of XDR was included in the study. Why did the authors write “XDR isolates” in this sentence?

- Thank you very much for the reminder. We revised the sentence accordingly.

2. Page 6, Result

*“Meropenem and amikacin had no bactericidal impact on intermediate and drug-resistant isolates; hence on all clinical isolates of A. baumannii in this study.”*

 According to Figure 1 and Table 1, not all concentrations of MEM + AK have no bactericidal effect on the clinical isolates. At twice MIC of this combination against MDR-1, it seems to provide ≥ 3 log _10_ reductions, which was considered to have bactericidal activity. Please rephrase this sentence. 

- Thank you for pointing this out. According to the colony count of the MDR-1 isolate (attached in the Underlying Data), the colony count decreased by 2.83 log 10 CFU/ml following exposure to 2 MIC + 2 MIC concentrations of meropenem and amikacin (from 5.78 log 10 CFU/ml to 2.95 log 10 CFU/ml). Because the reduction in colony counts did not surpass 3 log 10 CFU/ml over the period measured, we categorized the activity as bacteriostatic. We will attempt to add column in table with Δ Log information to ensure that the findings are more easily discernible.

3. Table 1

*“Bacteriostatic activity in the MEM 0.5 MIC + SAM 0.5 MIC against ATCC 19606 isolate, the MEM 2 MIC + SAM 0.5 MIC against MDR-1 isolate, the MEM 2 MIC + AK 2 MIC against MDR-1, and the MEM 1 MIC + SAM 1 MIC against XDR”*

 Please carefully verify the combination activity (bactericidal and bacteriostatic) in Table 1, because there are some incorrect results. it seems to provide ≥ 3 log10 reductions (bactericidal), compared to the growth control.

- Thank you for the comment. As stated in Point 2, the reduction in colony count for the isolates mentioned did not reach 3 log 10 CFU/ml; therefore, it was classified as bacteriostatic (MEM 0.5 MIC + SAM 0.5 MIC against ATCC 19606 isolate: 2.87 log 10 CFU/ml, MEM 2 MIC + SAM 0.5 MIC against MDR-1 isolate: 2.71 log 10 CFU/ml, MEM 2 MIC + AK 2 MIC against MDR-1: 2.83 log 10 CFU/ml, MEM 1 MIC + SAM 1 MIC against XDR 2.48 log 10 CFU/ml).  As it explains the growth of the isolates when exposed to antibiotics, the decrease in colony count of the isolates was compared to the time-to-time colony count of the isolates rather than to the growth control. We will attempt to add column in table with Δ Log information to ensure that the findings are more easily discernible.

Title and description of Table 1

- Thank you for the helpful reminder. We have made the necessary adjustments.

4. Data availability (Underlying Data)

 Recreate the supplementary tables (Colony Count Data and Turbidity Data)

- Revised accordingly.

**Minor Revision**

 1. Page 1, Abstract

*Results: “..… that were intermediate to ampicillin sulbactam and …”. *A hyphen between ampicillin sulbactam was missed. 

- Revised accordingly.

2. Page 2, Keywords

 “Acinetobacter baumannii” should be Italic

- We appreciate your pointing this out. Updated as required.

3. Page 3, Methods

*“… ATCC A.baumannii 19606 …” “… (CLSI) 2022 breakpoint for A.baumannii …”. *A space between genus and species was missed. 

- Thank you. Revised accordingly.

4. Page 3, Methods

*“… in a colony-forming unit (CFU)/mL over …”. *“mL” should be replaced by “ml” as same as the other part of the manuscript. 

- We concur.

5. Page 4, Results

*“In MDR 1 isolates, sc. MDR isolates that …”. *What does the “sc” means?

- Thank you for your inquiry. The word “sc” in the sentence above is an abbreviation of *scilicet*, a contraction of Latin *scire licet*, meaning "it is permitted to know." Sc. introduces additional information regarding something stated earlier, often in the form of a list to remove an ambiguity or supply a word omitted in the preceding text. We shall attempt to rephrase the sentence such that the meaning is more clearly apparent.

6. Page 4, Results

*“In MDR 1 isolates, sc. MDR isolates that were both carbapenem-resistant and intermediate to ampicillin-sulbactam”. *Rephrase the sentence.

- We concur.

7. Page 5, Results

*“At 24 and 48 hours, MDR 2 isolates (isolates resistant to meropenem and ampicillin-sulbactam) …”. *Use other codes such as “MDR-1” and “MDR-2”

- Thank you. Revised accordingly.

8. Page 6-7, Results

*“… these concentrations had no effect on the number of colonies in XDR isolates when compared to the number of colonies at 0 hours measurement.” *According to Figure 1, the authors should also specify the type of antibiotic combination, because this phenomenon was only found in XDR against MEM + AK combination, but not XDR against MEM + SAM. 

- We thank you for bringing this to our attention. As stated in Point 1.a, attempts are made to arrange the sentences.

9. Page 7, Discussion

*“… sulbactam has a high affinity for PBP 1 and 3” . *Replaced with “… sulbactam has a high affinity for PBP 1 and PBP 3”. 

- Revised accordingly.

10. Page 7, Discussion

*“Numerous investigations have demonstrated that subinhibitory concentrations of beta-lactam antibiotics can alter the shape of bacteria’s cell walls”. *Provide some references for this sentence.

- We have made adjustments in accordance with the revision.

11. Page 4 and 5, Figure 1 and Figure 2

 Use another colour and shape to represent the results of “Growth Control”

- We have made adjustments in accordance with the revision.

12. How many replications did the authors performed for time-kill assay?

- Experiments were conducted in six replications.

13. According to the description in Figure 1, Figure 2, and Table 1, why did the MICs not represent the exact MICs, but it represents the MIC at the susceptible breakpoint from CLSI guideline? 

- Thank you for the question. The antibiotic concentration was based on the CLSI breakpoint since the susceptible breakpoint value was based on the patient's clinically standard dosing regimen. This study aims to identify clinically relevant, effective antibiotic combinations for patients with MDR and XDR *A.baumannii *infections; consequently, it is necessary to utilize antibiotic concentrations achieved through a standard dosing regimen. MDR and XDR *A.baumannii* are frequently resistant to the tested antibiotic, with MIC typically being multiple times that of susceptible isolates, which is difficult to achieve during the administration of therapeutic antibiotic doses, thereby complicating the clinical application of those kinds of studies.
